# PD-0332991 induces G1 arrest of colorectal carcinoma cells through inhibition of the cyclin-dependent kinase-6 and retinoblastoma protein axis

**DOI:** 10.3892/ol.2014.1957

**Published:** 2014-03-10

**Authors:** CHUNSHENG LI, LING QI, ANITA C. BELLAIL, CHUNHAI HAO, TONGJUN LIU

**Affiliations:** 1 Department of Colorectal Surgery, The Third Hospital of Jilin University, Changchun, Jilin 130033, P.R. China; 2 Department of Pathology, Jilin Medical College, Jilin, Jilin 132013, P.R. China; 3 Department of Neurology and Neurosurgery, Montreal Neurological Institute, McGill University, Montreal H3A 2B4, Canada; 4 Department of Pathology, Montreal Neurological Institute, McGill University, Montreal H3A 2B4, Canada

**Keywords:** cell cycle, colorectal carcinoma, cyclin-dependent kinase-6, PD-0332991, retinoblastoma protein

## Abstract

Preclinical and clinical studies have demonstrated the anticancer activity of PD-0332991, a selective cyclin-dependent kinase 4/6 (CDK4/6) inhibitor, in the treatment of various types of cancer in a retinoblastoma protein (RB)-dependent manner. However, it remains unclear whether CDK4, CDK6 or both are required for RB phosphorylation in colorectal carcinoma and thus PD-0332991 can be used to target this CDK-RB axis for the cancer therapy. The aim of this study was to determine whether CDK4, CDK6 and phosphorylated RB proteins were overexpressed in colorectal carcinoma tissues as compared to matched normal colorectal tissues. The results showed that knockdown of CDK6 but not CDK4 reduced RB phosphorylation and inhibited carcinoma cell growth. Thus, CDK6 plays a critical role in RB phosphorylation and cancer growth. PD-0332991 treatment blocked RB phosphorylation and inhibited cell growth through the induction of G1 arrest of colorectal carcinoma cells. The results demonstrated that, by targeting of CDK6-RB axis, PD-0332991 may prove to be a novel therapeutic agent in treating colorectal carcinoma.

## Introduction

Excessive cell growth through the cell cycle is the fundamental hallmark of cancer (1). Cyclins and cyclin-dependent kinases (CDKs) drive the cell cycle progression from G1 to S phase and G2 to M phase (2). Of the four CDKs (CDK1, CDK2, CDK4 and CDK6), CDK4 and CDK6 are not required for the cell cycle of normal cells but are essential for driving the cell cycle progression in various types of cancer (3–5). It was previously reported that the selective targeting of CDK4/6 kinase activity may block the cell cycle and thus inhibit cancer growth (6). CDK4 and CDK6 interact with cyclin D and form the cyclin D/CDK4 and cyclin D/CDK6 complexes where CDK4/6 are activated for G1-S transition through phosphorylation of the retinoblastoma protein (RB) and its downstream E2F transcriptional factors (7).

The development of small molecule inhibitors targeting the cyclin-CDK4/6-RB axis for cancer therapy is crucial (8). However, the first generation of broad-range pan-CDK inhibitors such as flavopiridol (9) has not been of clinical benefit due to the toxicity and lack of specificity (10). Efforts have been focused on the next generation of CDK-specific inhibitors. PD-0332991 is a highly selective, orally administered and reversible inhibitor of CDK4/6 (11,12). PD-0332991 blocks the cell cycle of brain, breast, blood and pancreatic cancer cells in an RB-dependent manner (13–17). Treatment of PD-0332991 inhibits the growth of animal xenografts derived from these cancer cells (11,15,18,19). This selective CDK4/6 inhibitor is currently in phase I/II clinical trials for advanced cancers and earlier data from the trials have shown that PD-0332991 is well tolerated with a good safety profile in cancer patients (20–22). Based on the preclinical and clinical observations the potential of PD-0332991 for the treatment of colorectal carcinoma was investigated.

Colorectal carcinoma is the third most common type of cancer, but the second leading cause of cancer-related mortality (23). Thus, development of novel curative treatments for colorectal carcinoma is essential. The cyclin D family includes cyclins D1, D2 and D3. Cyclin D1 is known to be a predictive factor for therapeutic response of colorectal carcinoma whereas cyclin D2 is required for the CDK4/6-driven growth of colorectal adenoma cells (24). In addition, the E2F family protein E2F4 is involved in the cell cycle progression of colorectal carcinoma cells (25). Findings of those studies suggest the possible role of cyclin D-CDK4/6-RB axis in the growth of colorectal carcinoma. However, whether CDK4, CDK6 or both are required for the G1-S transition of colorectal carcinoma cells remains to be clarified.

In this study, we showed that CDK6 and RB are highly expressed in colorectal carcinoma tissues and derived cells as compared to the matched normal colorectal tissues. Both CDK6 and RB are required for the cell cycle progression of colorectal carcinoma cells and by inhibiting the CDK6-RB axis, PD-0332991 induces the G1 cell cycle arrest and inhibits cancer cell growth. Thus, PD-0332991 may be used for the treatment of colorectal carcinoma.

## Materials and methods

### Human colorectal carcinoma and matched normal tissues

Four human colorectal carcinoma and matched adjacent normal colorectal tissue samples were collected in the Third Hospital of Jilin University (Changchun, China) between January, 2010 and December, 2010, in accordance with the protocols approved by the Institutional Review Board of the Third Hospital of Jilin University. Patients provided written informed consent for the tissue collection. This study was approved by the Third Hospital Ethics Committee of Jilin University.

### Human colorectal carcinoma cell lines

The colorectal carcinoma cell lines CACO-2, COLO-205, COLO-320, DLD-1, HCT-8, HT29 and SW948, together with the human glioma cell line LN229 serving as the control, were purchased from the American Type Culture Collection (ATCC, Manassas, VA, USA). Each cell line was grown in RPMI-1640 medium (Invitrogen Life Technologies, Carlsbad, CA, USA) supplemented with 10% fetal bovine serum (FBS) and maintained in a humidified 37°C and 5% CO_2_ incubator (26).

### Reagents and antibodies

PD-0332991 was purchased from Selleckchem (Houston, TX, USA) and prepared as stock solutions in dimethyl sulfoxide (DMSO). Antibodies against CDK1, CDK4, CDK6, cyclin D1, cyclin D3, RB, phosphorylated-RB (pRB) (S780, S795 and S807/811) were purchased from Cell Signaling Technology, Inc., (Danvers, MA, USA). The antibody against CDK2 was purchased from Santa Cruz Biotechnology, Inc., (Santa Cruz, CA, USA) and β-actin antibody was purchased from Sigma-Aldrich (St. Louis, MO, USA). Horseradish peroxidase-conjugated goat anti-mouse and goat anti-rabbit antibodies were obtained from Jackson IR Laboratories, Inc., (West Grove, PA, USA). Protease inhibitor mixture, Triton X-100 and other chemicals were purchased from Sigma-Aldrich. The enhanced chemiluminescence detection kit was obtained from Amersham Biosciences (Piscataway, NJ, USA).

### Western blotting

Western blotting was performed as previously described (27). In brief, cell lines and tissues were lysed in lysis buffer consisting of 20 mmol/l Tris pH 7.4, 150 mmol/l NaCl, 1% NP-40, 10% glycerol, 1 mmol/l EGTA, 1 mmol/l EDTA, 5 mmol/l sodium pyrophosphate, 50 mmol/l sodium fluoride, 10 mmol/l β-glycerophosphate, 1 mmol/l sodium vanadate, 0.5 mmol/l DTT, 1 mmol/l PMSF, 2 mmol/l imidazole, 1.15 mmol/l sodium molybdate, 4 mmol/l sodium tartrate dihydrate and 1X protease inhibitor cocktail (Sigma, St. Louis, MO, USA). Following a 30-min incubation in lysis buffer at 4°C, lysates were centrifuged at 18,000 × g for 15 min at 4°C. The supernatant was collected and protein concentrations were determined by the Bradford protein assay following the manufacturer’s instructions (Bio-Rad, Hercules, CA, USA). Equal amounts of protein were separated through SDS-PAGE gels and transferred onto nitrocellulose membranes (Bio-Rad). The membranes were incubated overnight at 4°C with primary antibody and then for 1 h with horseradish peroxidase-conjugated secondary antibody. The membranes were developed by chemiluminescence.

### Lentiviral shRNA sequences and transduction

Lentiviral shRNA vectors were purchased from the Sigma MISSION^®^ shRNA library (Sigma-Aldrich) and included scrambled control (SHC002), CDK6-747 (TRCN0000039747, 5′-CGTGG AAGTTCAGATGTTGAT-3′) and CDK6-893 (TRCN000019 4893, 5′-CATGAGATGTTCCTATCTTAA-3′), CDK4-20 (TRCN0000010520, 5′-ACAGTTCGTGAGGTGGCTTTA-3′), and CDK4-64 (5′-ATGACTGGCCTCGAGATGTAC-3′). Each lentiviral shRNA vector was transduced into cells that were selected with puromycin as previously described (28).

### Cell proliferation assay

Cell proliferation was determined by acid phosphatase assay according to the manufacturer’s instructions (29,30). In brief, untreated or transduced cells with shRNA vectors were grown in 96-well plates at 1×10^3^ cells per well in 200 μl of 10% FBS-containing medium. Following incubation for 24 h, the medium was replaced with 10% FBS medium or the medium was supplemented with PD-0332991. After incubation for 1, 3 or 5 days, the cells were washed with phosphate-buffered saline (PBS) and each of the wells was added with 100 μl buffer containing 0.2 M sodium acetate (pH 5.5), 0.2% (v/v) Triton X-100 and 20 mmol/l p-nitrophenyl phosphate (Sigma 104 phosphatase substrate). The plates were incubated at 37°C for 1.5 h and the reaction was stopped by the addition of 10 μl 1 M NaOH to each well and staining was measured at 405 nm by a microplate reader (Bio-Rad).

### Flow cytometric analysis of the cell cycle

Flow cytometry was performed as previously described (26). In brief, cells were grown in 65-mm plates at a density of 5×10^5^ cells per well. After 24 h incubation, the cells were grown for 24 h in 10% FBS medium supplemented with or without PD-0332991 (1 μmol/l) in the presence or absence of DMSO as a control. After treatment, cells were collected, washed with PBS and fixed by incubation in 70% ethanol solution at 4°C. The fixed cells were washed and the cell pellets were stained using a propidium iodide-RNase solution (PBS containing 20 μg/ml propium iodide, 20 μg/ml DNase-free RNase A and 0.1% Triton X-100) for 30 min at 20°C in the dark. The cell cycle status was analyzed with a flow cytometer using FlowJo software (Tree Star, Inc., Ashland, OR, USA).

### Statistical analysis

Data were presented as the means ± standard deviation (SD) and analyzed statistically by Student’s t-test. P<0.05 was considered statistically significant.

## Results

### CDK proteins are overexpressed in human colorectal carcinoma

**Figure 1:**
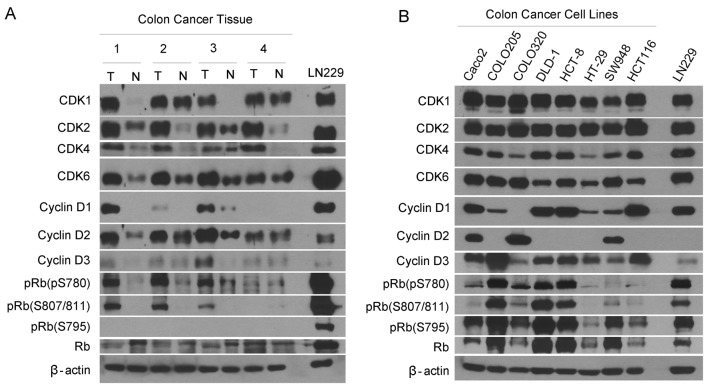
Cell cycle proteins are overexpressed in human colorectal carcinoma. (A) Western blotting of human colorectal carcinoma tissues (T) and matched adjacent normal colorectal tissues (N) (above the panel) of the expression of the cell cycle proteins (left of the panel). (B) Western blot analysis of the expression of these cell cycle proteins (left of the panel) in eight colorectal carcinoma cell lines (above the panel). The glioblastoma cell line LN229 was included as the positive control and β-actin (Actin) was used as protein loading control. CDK, cyclin-dependent kinases; pRB, phosphorylated retinoblastoma protein.

To the best of our knowledge, this is the first study concerning the expression of cell cycle proteins in colorectal carcinoma tissues. We examined the expression of key cell cycle proteins in surgically resected colorectal carcinoma tissues as compared with matched adjacent normal colorectal tissues. Western blotting revealed that CDK1, CDK2, CDK4 and CDK6 proteins were expressed at much higher levels in the carcinoma tissues than the matched normal tissues (Fig. 1A). These CDK proteins were expressed in the carcinoma-derived cell lines (Fig. 1B). Cyclin D1 was detected in half of the carcinoma tissues and cyclin D3 was observed in only one of the four carcinoma tissues, while cyclin D1 and D3 were slightly detected in normal tissues. By contrast, cyclin D2 was expressed in all the carcinoma and matched normal tissues with the expression levels being higher in the carcinoma tissues. Consistent with this profile, cyclin D1 was highly expressed in four but weakly in three; cyclin D2 was highly expressed in three and cyclin D3 was expressed in seven of eight cell lines.

We examined the expression of unphosphorylated RB and pRB. RB can be phosphorylated at several serine residues including S780, S795, S807 and S811 (31). Thus, the antibodies against pRB (S780), pRB (S795) and pRB (S808/811) were used in this study. Western blotting detected pRB (S780) and pRB (S808/811) in three of four carcinoma tissues but only slightly in the normal tissues (Fig. 1A). The pRB (S795) was observed only in the cell lines but not any tissues as compared with the expression in the control glioblastoma cell line LN229. By contrast, pRB (S795) was detected in all the cell lines (Fig. 1B). Collectively, the G1 cyclins, CDK and pRB are expressed differentially in human colorectal carcinoma tissues and cell lines.

### PD-0332991 treatment induces G1 arrest in colorectal carcinoma cells

**Figure 2:**
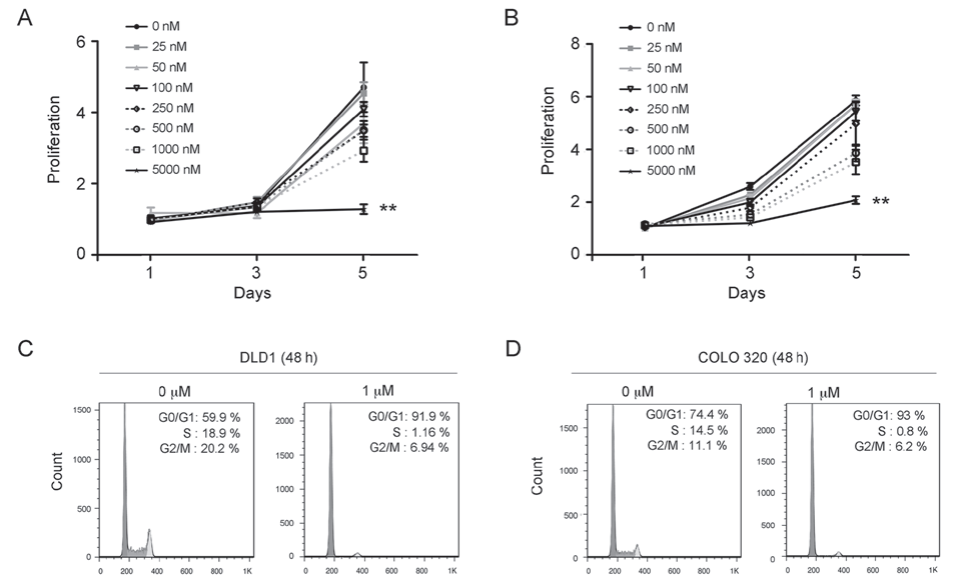
PD-0332991 inhibits cell growth through the induction of G1 cell cycle arrest. The colorectal carcinoma cell lines (A) DLD-1 and (B) COLO320 were treated with various doses of PD-0332991 for the days as indicated (bottom of the panel) and examined by cell proliferation assay. The experiments were repeated three times and data are presented as the mean ± standard deviation and analyzed statistically by the Student’s t-test. ^**^P<0.001. (C) DLD-1 and (D) COLO320 cells were also treated with 1 μM of PD-0332991 for 48 h and analyzed by flow cytometry for the cell cycle. G0/G1, S and G2/M phase cells were presented as a percentage.

To explore the potential of PD-0332991 in treating colorectal carcinoma, we examined whether the carcinoma cells respond to PD-0332991 treatment in culture. The carcinoma cell lines DLD-1 and COLO320 were treated with PD-0332991 concentrations ranging between 25 nM and 5000 nM for 72 h. Results of the cell proliferation assay showed that PD-0332991 treatment significantly inhibited the growth of DLD-1 (Fig. 2A) and COLO320 cells in a dose-dependent manner (Fig. 2B). To evaluate whether inhibition by PD-0332991 occurs through the cell cycle, DLD-1 and COLO320 cells were treated or untreated with PD-0332991 (1 μM) for 48 h and subjected to flow cytometry for the cell cycle as previously described (30). The results showed that the PD-0332991 treatment led to a significant increase in the number of G1 phase cells but a marked decrease of the S phase cells in the DLD-1 (Fig. 2C) and COLO320 cells (Fig. 2D). These findings suggest that PD-0332991 treatment inhibits growth through the induction of G1 arrest of colorectal carcinoma cells.

### PD-0332991 treatment inhibits RB phosphorylation in colorectal carcinoma cells

**Figure 3:**
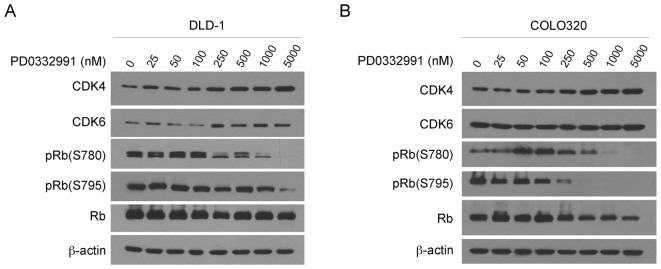
PD-0332991 blocks RB phosphorylation in colorectal carcinoma cells. (A) The DLD-1 and (B) COLO320 cells were treated at various concentrations of PD-0332991 as indicated (top of the panel) for 48 h. The total proteins were extracted from these treated and untreated cells (controls) and subjected to western blotting using the antibodies as indicated (left of panel). CDK, cyclin-dependent kinases; pRB, phosphorylated retinoblastoma protein.

To examine the mechanisms in the PD-0332991-induced G1 arrest, we examined the CDK4/6 and RB proteins in DLD-1 and COLO320 cells after 24 h treatment with a series of concentrations of PD-0332991. Western blotting revealed that the treatment did not affect the levels of CDK4, CDK6 and unphosphorylated RB in DLD-1 (Fig. 3A) and COLO320 cells (Fig. 3B). By contrast, PD-0332991 treatment markedly reduced the levels of pRB (S780) and pRB (S795) in each of the cell lines in a dose-dependent manner. Collectively, PD-0332991 treatment inhibits RB phosphorylation, induces G1 arrest and thus suppresses the growth of colorectal carcinoma cells in culture.

### CDK6 phosphorylates RB for the growth of colorectal carcinoma cells

**Figure 4:**
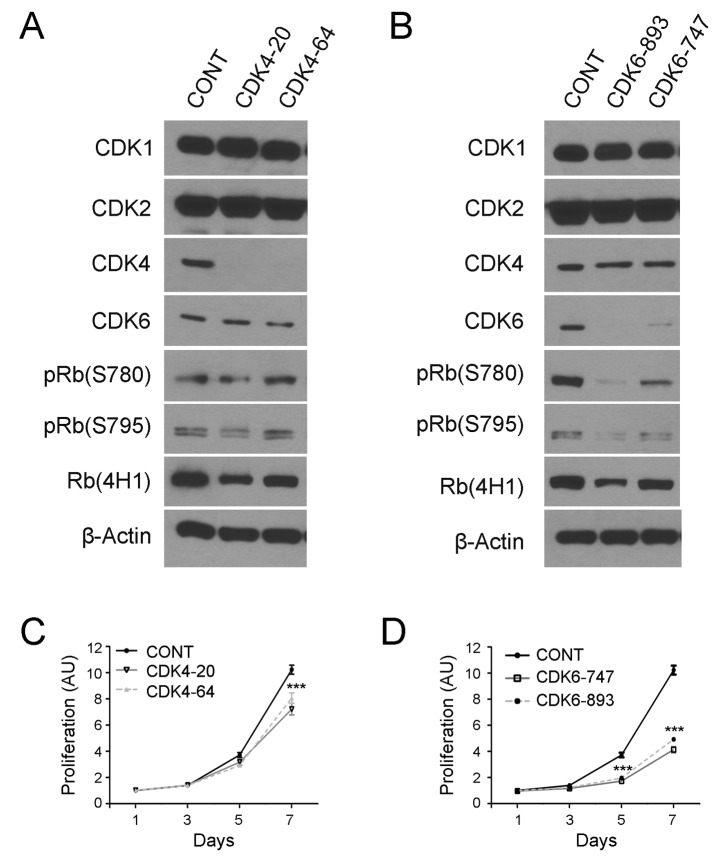
CDK6 phosphorylates RB for cancer cell growth. (A) COLO320 cells were transduced with lentiviral vectors encoding CDK4 shRNA sequences (CDK4-20, CDK4-64) and (B) CDK6 shRNA sequences (CDK6-893, CDK6-747) and were examined by western blotting using the antibodies as indicated (left of panel). The scrambled shRNA-encoded vector was used as the control (CONT) in the two experiments. (C) The cells transduced with CDK4-shRNA and (D) CDK6-shRNA vector were examined by a cell proliferation assay. The data show the mean ± standard deviation of three independent experiments. Student’s t-test was used to analyze the data. ^**^P<0.001. CDK, cyclin-dependent kinases; pRB, phosphorylated retinoblastoma protein.

CDK4 and CDK6 regulate the G1-S cell cycle transition (2). However, whether CDK4 or CDK6 or both were required for RB phosphorylation and G1-S transition in colorectal carcinoma cells remained to be clarified. To address this issue, lentiviral vectors encoding CDK4/CDK6-specific shRNA sequences were transduced into COLO320 cells as previously described (28). To prevent off-target effects, two shRNA target sequences were used for each of the kinases including CDK4-20 and CDK4-64 to target CDK4 and CDK6-747 and CDK6-893 for CDK6 knockdown. The transduced cells were examined by western blotting and the results revealed that the transduction of the CDK4 and CDK6 shRNA encoding vectors eliminated the expression of CDK4 (Fig. 4A) and CDK6 protein (Fig. 4B), respectively, in COLO320 cells.

Notably, CDK4 knockdown did not affect RB phosphorylation as evidenced by western blotting using the pRB (S780) and pRB (S795) antibodies (Fig. 4A). By contrast, CDK6 knockdown markedly reduced the expression of these phosphorylated RB proteins in the carcinoma cells (Fig. 4B). To examine the effects of CDK4/6 knockdown and RB phosphorylation inhibition on cancer cell growth, we transduced the shRNA-coded vectors in COLO320 cells, selected stably transduced cells and examined cell growth using a cell viability assay. The results showed that the knockdown of CDK4 slightly inhibited the growth of COLO320 cells (Fig. 4C), whereas the knockdown of CDK6 resulted in a marked inhibition of the growth of COLO320 cells (Fig. 4D). The data suggest that CDK6 plays a critical role in RB phosphorylation and cell growth in colorectal carcinoma cells.

## Discussion

Colorectal carcinoma is the second leading cause of cancer-related mortality due to the lack of curative treatments (23). Previously, signaling pathways were found to be involved in the formation and progression of colorectal carcinoma for the development of cancer signal pathway-targeted therapies. However, such targeted therapies have not been materialized for the effective treatment of this lethal cancer (32). Novel therapeutic agents are therefore required for treatment of this type of cancer. In the present study, the therapeutic potential of the selective CDK4/6 inhibitor PD-0332991 in treating colorectal carcinoma was demonstrated.

The cyclin D-CDK4 and cyclin-CDK6 complex drives the cell cycle through G1-S transition via phosphorylation of RB in various types of cancer (2). Thus, therapeutic agents have been generated targeting these G1 phase kinases for cancer therapies (6). Of these novel therapeutic agents, the CDK4/CDK6 selective and potent inhibitor PD-0332991 (11) has passed the safety test with anticancer activity in phase I/II trials (20–22). However, there are currently no studies regarding the therapeutic potential of this inhibitor in the treatment of colorectal carcinoma. Similarly, there are few studies with regard to the cell cycle pathway in this cancer.

The data presented herein have shown that the G1 phase cyclin D1, D2, D3, CDK4, CDK6 and pRB proteins are highly expressed in colorectal carcinoma tissues as compared to the matched adjacent normal colorectal tissues. CDK4 and CDK6 control the G1-S transition through the cell cycle (2) and recent studies of genetically-engineered mice suggest that CDK4 or CDK6 is used to drive the cell cycle in each type of cancer (3–5). To identify the G1 kinase in colorectal carcinoma, we have shown that knockdown of CDK6 but not CDK4 markedly reduces RB phosphorylation and inhibits the growth of colorectal carcinoma cells, suggesting for the first time that CDK6-RB axis is crucial in the growth of the carcinoma and targeting of the CDK6-RB axis may provide a novel therapeutic strategy in the treatment of colorectal carcinoma.

Preclinical studies have demonstrated the anticancer activities of PD-0332991 in treating brain, breast, blood and pancreatic cancers in an RB-dependent manner (13–17). To the best of our knowledge, this study has shown for the first time that PD-0332991 treatment blocks RB phosphorylation, induces G1 arrest and thus inhibits the growth of human colorectal carcinoma cells. This study therefore suggests that PD-0332991 has therapeutic effects against colorectal carcinomas through inhibition of the CDK6/RB pathway. Thus, cell cycle pathways in colorectal carcinoma and the development of PD-0332991 into an effective therapeutic agent for clinical treatment of human colorectal carcinoma should be investigated.
